# Analysis of Masseter Muscle Activity Following Treatment of Fully Edentulous Patients With Mono-arch and Bi-arch All-on-4 Prostheses: A Systematic Literature Review

**DOI:** 10.7759/cureus.92848

**Published:** 2025-09-21

**Authors:** Mohamad Awos Sulaiman, Albertas Kriauciunas

**Affiliations:** 1 Odontology, Lithuanian University of Health Sciences, Kaunas, LTU; 2 Clinic of Dental and Maxillofacial Orthopedics, Lithuanian University of Health Sciences/Hospital of Lithuanian University of Health Sciences, Kaunas, LTU

**Keywords:** all-on-4 concept, bi- and mono-arch all-on-4, edentulous maxillary and mandibular arches, edentulous patients, fixed prosthodontics, masseter, removable prosthodontics

## Abstract

In the current modern era, edentulism of multifactorial origin remains a widespread condition with a rapidly increasing annual prevalence. This condition impairs various components of the stomatognathic system, particularly the masticatory muscles, with the masseter muscle being the most notably affected. However, ongoing innovations in surgical prosthodontics have made the All-on-4 (AO4) prosthetic concept a sustainable and minimally invasive option for the rehabilitation of fully edentulous patients, showing promising improvements in oral function. The objective of this systematic review was to analyze existing research on changes in masseter muscle activity in fully edentulous patients treated with mono- and bi-arch AO4 prostheses, assessed through electromyography (EMG), bite force, and occlusal contact analysis. The review followed the Preferred Reporting Items for Systematic Reviews and Meta-Analyses (PRISMA) 2020 guidelines, and the research question was formulated using the population, intervention, control, and outcomes (PICO) framework: “How is the activity of the masseter muscle affected in fully edentulous patients rehabilitated with mono- and bi-arch AO4 prostheses?” According to the inclusion and exclusion criteria, articles were sourced from PubMed, Google Scholar, and ScienceDirect. All included studies were published within the past five years. Risk of bias was assessed using the Cochrane Risk-of-Bias 2 (RoB-2) tool. Out of 447 screened articles, only three met the inclusion criteria, comprising a total of 37 patients. Two studies focused on mono-arch and one on bi-arch AO4 prostheses. All three studies consistently demonstrated increased masseter muscle activity in both mono- and bi-arch AO4 groups compared to other prosthetic rehabilitation options, such as two-implant-supported overdentures and conventional dentures (CDs). However, bi-arch AO4 prostheses demonstrated superior masseter muscle neuromuscular activity compared to mono-arch AO4. Nevertheless, the type of prosthesis fixation (fixed or overdenture) and the material used (porcelain fused to metal or acrylic resin) may influence masseter muscle EMG analysis. Understanding these prosthetic influencing factors in mono- and bi-arch AO4 prostheses will help clinical practitioners rehabilitate fully edentulous patients more effectively.

## Introduction and background

Edentulism is described as a universal condition of lacking all natural teeth [1]. In the current era, approximately 353 million people suffer from edentulism of multifactorial origin, with an annual increase of 5.11% [2]. Given the increasing occurrence, edentulism’s etiology is considered multifactorial, ranging primarily from microbial and genetic factors to secondary causes, including iatrogenic, traumatic, and therapeutic factors [3]. Additionally, socioeconomic status, education, and poor oral and general health have been identified as additional causative factors for eventual edentulism [4]. 

Beyond the complex etiology, edentulism results in various oral health consequences. For instance, it disrupts oral mucosal protective functions due to reduced tissue regeneration and resistance [5]. This is often combined with salivary gland alterations in edentulous patients, including decreased salivary flow and pH, along with increased viscosity, which interferes with mucosal hydration and further escalates the risk of oral mucosal diseases (e.g., stomatitis, candidiasis) [6]. Most significantly, edentulism leads to pathological bone resorption due to the absence of physiological bony stimulation generated by the periodontal ligaments during mastication [7].

More specifically, regarding chewing function, the masseter muscle is one of the primary and most significant muscles of the masticatory system, with an essential role in elevating the mandible to generate bite force during mastication, as well as contributing to protrusion and lateral movements [8]. However, with the complete absence of teeth, the masseter muscle undergoes functional and morphological alterations. For instance, several studies [5,9,10] have confirmed a significant reduction in masseter muscle thickness in edentulous individuals due to their inability to function as vigorously as in dentate individuals, leading to diminished bite force. Moreover, edentulous patients wearing full conventional dentures (CDs) demonstrate only one-fourth to one-fifth the bite force of dentate individuals and require seven additional chewing strokes to reduce food to half its size [11,12]. Nevertheless, few studies have emphasized that the number of teeth and occlusal contacts is also linked to the severity of overall masticatory functional efficacy in patients experiencing progressive edentulism or undergoing full-arch prosthetic rehabilitation [13,14]. Yet, the impact of edentulism rehabilitation on neuromuscular function remains insufficiently addressed in the current literature.

At present, when rehabilitating edentulous patients for optimal quality of life, full-arch implant-supported prostheses are usually preferred over complete dentures due to higher patient satisfaction in terms of stability, retention, comfort, and biocompatibility [15]. Despite the advantages, conventional full-arch implant-supported prostheses are not always an immediately reliable solution for all patients. For instance, the previously mentioned consequence of edentulism, bone resorption, particularly in the posterior ridges, often requires patients to undergo supplementary bone grafting and sinus lifting procedures, followed by a healing period to establish sufficient bone for future implant placement [16,17]. However, in 2003, Paulo Malo introduced the All-on-4 (AO4) concept as a solution to bypass these limitations. The concept involves placing two anterior implants in an axial direction and two posterior implants at an angle of up to 45 degrees toward the distal aspect, effectively avoiding unfavorable posterior bony areas and eliminating the need for additional invasive preoperative surgical procedures for edentulous patients [18].

Despite intense debate over the AO4 concept and the currently available data, no previous systematic review has examined its neuromuscular sequelae. Therefore, this review aims to consolidate all available evidence on how rehabilitation with mono- and bi-arch AO4 prostheses affects the masseter muscle following placement in fully edentulous patients. The main examination method is electromyography (EMG), which measures muscular activity through conversion into interpretable electrical signals [19]. Additional tools, such as bite force devices to record the occluding force produced by the masticatory muscles and a T-scan device to observe the occlusal contact relationship between the teeth of both arches, were also used [20,21].

Particularly, the three clinical trials reporting surface EMG output of AO4 published within the last five years provide varied results depending on arch modality, fixation, and material. Previous full-arch reviews have focused on complications or survival, excluding muscular activity. Thus, we formulated a research question: “How is the activity of the masseter muscle affected in fully edentulous patients rehabilitated with mono- and bi-arch AO4 prostheses?” Addressing this gap is crucial, since the masseter muscle governs bite force, chewing efficiency, and peri-implant load.

## Review

Materials and methods 

Aim of the Systematic Review

This systematic review aimed to assess changes in masseter muscle activity in fully edentulous patients following treatment with mono- and bi-arch AO4 prostheses, based on EMG, bite force, and occlusal contact assessments. 

Inclusion and Exclusion Criteria 

To ensure methodological rigor, we predefined a comprehensive set of inclusion and exclusion criteria (Table 1 and Table 2). 

**Table 1:** Inclusion criteria. EMG, electromyography; AO4, All-on-4

Article inclusion criteria
Articles analyzing EMG activity of the masseter muscle, occlusal contact relationship, or bite force at one or multiple time points (i.e., before, immediately after, or following a period) after mono- or bi-arch AO4 prosthesis placement in fully edentulous patients
Articles treating fully edentulous patients according to the AO4 implantation protocol
Articles including patients aged 50 to 65 years who are free from infections, inflammations, systemic diseases (including cardiovascular, skeletal, endocrine, hepatobiliary, autoimmune, neuromuscular conditions), temporomandibular joint disorders, and smoking habits
Full-text, English-language journal articles published from 2020 to 2025

**Table 2:** Exclusion criteria. EMG, electromyography; AO4, All-on-4

Article exclusion criteria
Articles not analyzing masseter muscle activity by means of EMG, bite force, occlusal contacts, or any other methodology in fully edentulous patients rehabilitated with mono- or bi-arch AO4 prostheses
Articles not including fully edentulous patients
Articles treating fully edentulous patients using a system other than AO4 and its protocol
Articles lacking comprehensive details or results on masseter muscle activity and the implantation protocol used
Articles including patients outside the 50-65 age range or those affected by infections, inflammations, systemic diseases (e.g., cardiovascular, skeletal, endocrine, hepatobiliary, autoimmune, and neuromuscular conditions), temporomandibular joint disorders, or smoking habits
Case reports and series, book chapters, theses, literature reviews, systematic reviews, and meta-analyses
Non-full-text articles written in a language other than English or not published between 2020 and 2025

Information Sources and Search Strategy 

This systematic review acquired all available clinical trial data published between 2020 and 2025, following the Preferred Reporting Items for Systematic Reviews and Meta-Analyses (PRISMA) guidelines [22]. Scientific database search engines, including PubMed, Google Scholar, and ScienceDirect, were used to identify the included clinical studies. The searches were conducted using Boolean keyword combinations: “masseter” AND “edentulous” OR “edentulism” AND “all-on-4” OR “all-on-four” OR “all on four” OR “all on 4”.

Additionally, a question framework was formulated according to the population, intervention, control, and outcomes (PICO) study design protocol: “How is the activity of the masseter muscle affected in fully edentulous patients rehabilitated with mono- and bi-arch AO4 prostheses?”.

The definitive included articles underwent five stages: 1. identification of articles by title relevance, 2. manual and automated removal of duplicates, non-English, and non-journal articles, 3. inclusionary screening based on title and abstract relevance, 4. full-text access verification, and 5. inclusionary screening based on full-text relevance.

The titles, abstracts, and full texts were carefully screened and analyzed by two authors (Sulaiman MA and Kriaučiūnas A), according to predefined inclusion and exclusion criteria. After a detailed analysis of the included studies, their risk of bias was evaluated using the Cochrane Risk of Bias-2 (RoB-2) tool protocol [23]. 

Study Selection 

**Figure 1:**
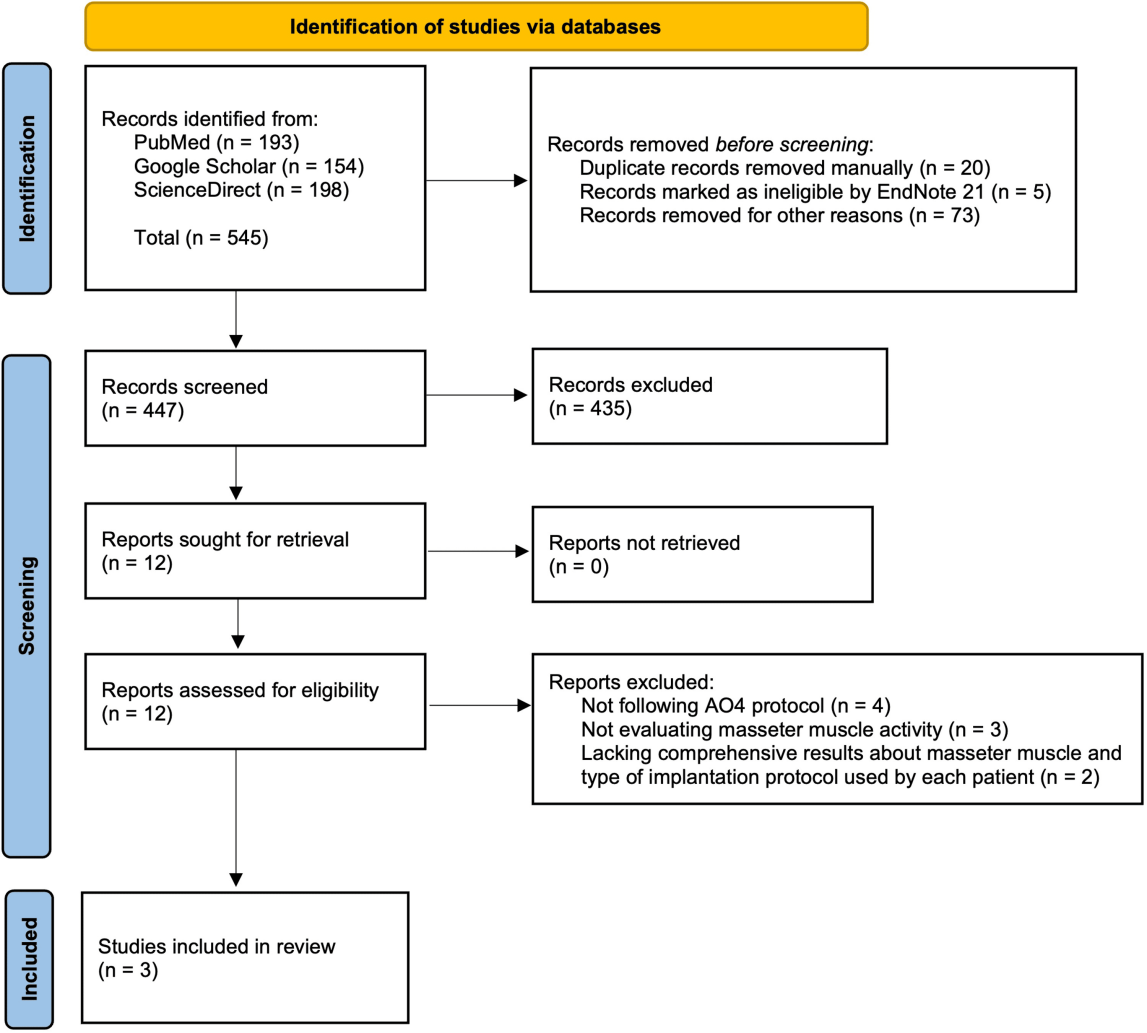
PRISMA flow diagram of the study selection process. PRISMA, Preferred Reporting Items for Systematic Reviews and Meta-Analyses

The included clinical trial studies were extracted from databases in accordance with the PRISMA 2020 statement guidelines, specifically illustrated in a flow diagram for systematic reviews with a database search chart only. During the identification phase, a total of 545 results were retrieved using Boolean keyword combinations in each database (PubMed, Google Scholar, and ScienceDirect). Before screening, 25 duplicates were removed using an automation tool (EndNote 21) and manual review. Additionally, 73 records were excluded for being non-English or non-journal articles. This left 447 records for the first step of the screening phase, of which 435 were excluded based on the inclusion and exclusion criteria. A total of 12 articles proceeded to the second step of screening, where all were assessed for full-text availability and found to be accessible. In the third step of the screening phase, all 12 articles underwent full-text eligibility assessment based on the inclusion and exclusion criteria. Of those, the following were excluded: four articles for not following the AO4 protocol, three articles for not evaluating masseter muscle activity, and two articles for lacking comprehensive details and results on masseter muscle activity or the type of implantation protocol used for each patient. In total, three clinical studies were deemed eligible for inclusion in the systematic review. Figure 1 illustrates the PRISMA flow diagram of the study selection process.

Characteristics of the Studies 

The first study conducted a comparative analysis of masseter muscle activity and occlusal contacts in seven fully edentulous subjects aged 50 to 65 years who were rehabilitated with mono-arch (mandibular) AO4 prostheses while retaining a CD in the maxilla. The authors assessed both the acrylic resin provisional and porcelain-fused-to-metal (PFM) definitive prosthetic stages of the mandibular AO4 during the treatment process [24]. Masseter muscle activity was measured using EMG, and occlusal contacts were assessed using a T-Scan device.

The second study included 18 fully edentulous participants (nine men and nine women) with an average age of 58.7±3.1 years, who underwent a comparative EMG evaluation of masseter muscle activity. The analysis was conducted first with full CDs and later with two types of mono-arch (mandibular) AO4 prostheses: fixed prostheses (FPD) and milled bar overdentures (MBO), while the maxilla remained with a CD [25].

The third study included 12 fully edentulous subjects, divided equally into two groups. The first group, with a mean age of 55.8 years, had only the mandible restored with a two-implant-supported mandibular overdenture and a CD in the maxilla, while the second group, with a mean age of 52.6 years, was restored with bi-arch AO4 prostheses [26]. Both groups subsequently underwent bite force measurements using an electromechanical bite force transducer and an EMG chewing efficiency analysis of the masseter, temporalis, and anterior digastric muscles.

It is worth noting that two studies [24,26] conducted bilateral masseter muscle EMG analysis, while one study [25] analyzed only the masseter muscle on the preferred chewing side. Meanwhile, masseter muscle EMG analysis in two studies [24,25] was performed using a food mastication protocol with two consistencies (soft and hard), while one study [26] used three food consistencies (soft, medium, and hard). Nevertheless, all three studies [24-26] had similar surface electrode placement on the masseter muscle, except for the ground electrodes: in two studies [24,26], they were placed on the forehead, while in one study [25], on the chin. In total, 37 subjects were included across all three studies.

Types of Studies 

The systematic review includes three studies, two within-subject crossover clinical studies [24,25] and one in-vivo comparative clinical study [26].

Risk of Bias Assessment Within Studies 

**Figure 2:**
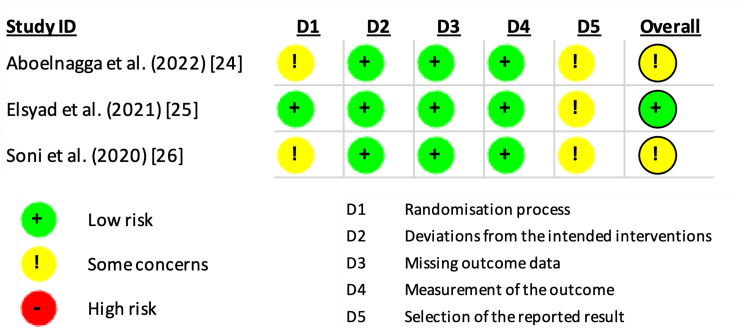
Cochrane RoB-2 assessment results of the three included studies, with bias evaluated for each domain and overall. RoB-2, Risk-of-Bias 2

**Figure 3:**
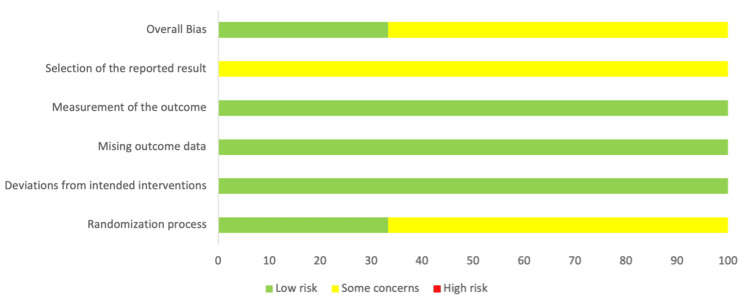
Summarized bias assessment results of the three included studies using the Cochrane RoB-2 tool. RoB-2, Cochrane Risk-of-Bias 2

The assessment of the risk of bias in the selected studies was conducted according to the guidelines outlined by the Cochrane RoB-2 tool [20]. The analysis illustrated that one study [22] had a low risk of bias in the randomization process (D1), while the other two [21,23] demonstrated some concerns. All three included studies [21-23] had a low risk of bias in the domains of deviations from the intended intervention (D2), missing outcome data (D3), and measurement of the outcome (D4), but raised some concerns regarding the selection of reported results (D5). As a result, two studies [21,23] were categorized as having an overall risk of bias of some concerns, while one study [22] was categorized as low risk (Figure 2 and Figure 3). 

Certainty of Evidence Analysis

The assessment of evidence certainty was conducted according to the Recommendations Assessment, Development, and Evaluation (GRADE) framework [27]. This method evaluates five domains: risk of bias, inconsistency, indirectness, imprecision, and other conditions, rating them as high, moderate, low, or very low. The analysis rated most outcomes as low due to risk of bias and serious imprecision, and very low for occlusal contact because of very serious imprecision. The GRADE certainty-of-evidence table is illustrated in Table 3.

**Table 3:** Certainty of evidence analysis using the GRADE framework. GRADE, Grading of Recommendations, Assessment, Development, and Evaluation

Study	Outcome	Number of studies	Number of patients	Study design	Risk of bias	Inconsistency	Indirectness	Imprecision	Other conditions	Certainty
Aboelnagga et al. [24], 2022 and Soni et al. [26], 2020	Masseter muscle activity	2	19	Observational	Serious	Not serious	Not serious	Serious	None identified	Low
Aboelnagga et al. [24], 2022	Occlusal contact	1	7	Observational	Serious	Not serious	Not serious	Very serious	None identified	Very low
Elsyad et al. [25], 2021	Amplitude	1	18	Observational	Not serious	Not serious	Not serious	Serious	None identified	Low
Elsyad et al. [25], 2021	Chewing rate	1	18	Observational	Not serious	Not serious	Not serious	Serious	None identified	Low
Elsyad et al. [25], 2021	Time of masticatory cycle	1	18	Observational	Not serious	Not serious	Not serious	Serious	None identified	Low
Elsyad et al. [25], 2021	Time of masticatory burst	1	18	Observational	Not serious	Not serious	Not serious	Serious	None identified	Low
Elsyad et al. [25], 2021	Masticatory time	1	18	Observational	Not serious	Not serious	Not serious	Serious	None identified	Low
Soni et al. [26], 2020	Bite force	1	12	Observational	Serious	Not serious	Not serious	Serious	None identified	Low

Statistical Procedures 

Considering the three clinical trials with varying EMG protocols, a quantitative meta-analysis would have yielded inconsistent heterogeneity statistics and potentially unreliable pooled estimates. In accordance with SWiM (Synthesis Without Meta-analysis) guidelines, we employed a structured narrative synthesis. 

Results 

Studies Based on Mono-arch AO4 Treatment 

The study conducted by Aboelnagga et al. revealed a higher masseter muscle activity when using acrylic resin provisional prostheses compared to PFM definitive prostheses during the mastication of both soft (P<0.0001) and hard (P<0.0001) food. Additionally, higher activity was observed during the mastication of hard food compared to soft food in both acrylic resin provisional (P<0.0001) and PFM definitive (P=0.0003) prostheses [24]. Furthermore, it was noted that EMG and T-scan demonstrated a weak, positive, and statistically insignificant correlation in soft acrylic resin provisional prostheses (P=0.96) and a weak, negative, and insignificant correlation in hard acrylic resin provisional prostheses (P=0.01). In contrast, soft and hard food with PFM definitive prostheses showed a moderate, positive, and insignificant correlation (P=0.25 and P=0.21, respectively) [24]. 

Additionally, it is worth mentioning that the T-scan revealed a significant imbalance in force distribution between the right and left sides in acrylic resin provisional prostheses (P<0.0001), while PFM definitive prostheses (P<0.0001) demonstrated only a minimally significant imbalance. Overall, the imbalance showed that forces produced on the right side were higher than on the left side in both prostheses (P<0.0001) [24].

In the study by Elsyad et al., the masseter muscle amplitude analysis showed that MBO had the highest amplitude, followed by FPD, and CD as the lowest, with a significant difference during the mastication of both soft (P=0.007) and hard (P=0.003) food. All prostheses exhibited higher masseter muscle activity during the chewing of hard food (P=0.043, 0.008, 0.043 for CD, FPD, and MBO, respectively) [25]. Regarding chewing rate assessment, CD showed the highest rate, followed by FPD, and MBO as the lowest, with a notable difference between soft (P=0.008) and hard (P=0.002) food. Additionally, all prostheses presented a higher chewing rate for hard food compared to soft food (P=0.042, 0.043, 0.049 for CD, FPD, and MBO) [25]. Concerning the masticatory cycle time, MBO was the longest, followed by FPD, and CD was the shortest time, with a significant difference between soft (P=0.009) and hard (P=0.004) food. However, all prostheses showed a longer chewing cycle time for soft food (P=0.049, 0.043, 0.048 for CD, FPD, and MBO) [25]. For masticatory burst time, MBO was the longest, followed by FPD, and CD was the shortest, demonstrating a significant difference between soft (P=0.012) and hard (P=0.008) food. Notably, the burst time for hard food was higher than for soft food in all prostheses (P=0.041, 0.043, 0.043 for CD, FPD, and MBO) [25]. Subsequently, masticatory time was the longest for CD, while FPD and MBO showed shorter durations. A significant difference between soft (P=0.014) and hard (P=0.008) food was seen. Furthermore, all prostheses showed a more pronounced masticatory time for hard food (P=0.042, 0.039, 0.042 for CD, FPD, and MBO) [22]. Finally, across all the analyses, no statistically significant differences were observed between MBO and FPD (P>0.05) [25]. *Study Based on Bi-arch AO4 Treatment *

In the final study by Soni et al., chewing efficiency EMG analysis demonstrated significantly higher bilateral masseter muscle activity for the AO4 prosthesis compared to the two-implant-supported OD during the mastication of soft (P=0.03, 0.06 for right and left sides), medium (P=0.038, 0.114), and hard (P=0.045, 0.051) food [26]. Similar findings were observed in comparison between AO4 prosthesis and CD for soft (P=0.003, 0.004), medium (P=0.011, 0.002), and hard (P=0.016, 0.002) food [26]. Overall, the AO4 prosthesis demonstrated the highest masseter muscle activity, followed by two-implant-supported OD, with CD as the lowest across all food consistencies. Moreover, regarding bite force, the AO4 prosthesis revealed significantly higher values than OD (P=0.016, 0.005), followed by CD (P=0.008, 0.002) [26]. The PICO table of masseter muscle activity analyses is presented in Table 4. 

**Table 4:** PICO analysis table of the included studies. PICO, population, intervention, control, and outcomes; AO4, All-on-4; CD, conventional dentures; FPD, fixed prostheses; MBO, metal bar overdenture; OD, overdenture; PFM, porcelain-fused-to-metal

Study	Population	Intervention	Comparison	Outcome
Aboelnagga et al. [24], 2022	N=7; fully edentulous	Mono-arch (mandibular) AO4 acrylic resin provisional and PFM definitive prostheses, with a CD in the maxilla	Effect of acrylic resin provisional and PFM definitive AO4 prostheses on occlusal contacts and masseter muscle activity during mastication of soft and hard food	Higher masseter muscle activity during use of the acrylic resin provisional prosthesis and mastication of hard food. Greater occlusal contact imbalance in the acrylic resin provisional prosthesis, with higher force produced on the right side in both prostheses. Overall, no significant correlation between occlusal contacts and masseter muscle activity was detected.
Elsyad et al. [25], 2021	N=18; two groups: fully edentulous, within-subject crossover (n=9 each)	CD, followed by mono-arch (mandibular) FPD and MBO AO4, with CD in the maxilla	Effect of CD, AO4 FPD, and MBO prostheses on masseter muscle activity during mastication of soft and hard food, based on five parameters: amplitude, chewing rate, time of masticatory cycle, time of masticatory burst, and masticatory time	Amplitude (highest to lowest): MBO, FPD, and CD. Chewing rate (highest to lowest): CD, FPD, and MBO. Time of masticatory cycle (longest to shortest): MBO, FPD, and CD. Time of masticatory burst (longest to shortest): MBO, FPD, and CD. Masticatory time (longest to shortest): CD, FPD, and MBO. Overall, no significant difference was observed between MBO and FPD in all five parameters.
Soni et al. [26], 2020	N=12; two groups: fully edentulous (n=6 each)	Group one: CD, followed by mono-arch (mandibular) two-implant-supported OD and CD in the maxilla. Group two: CD, followed by bi-arch AO4	Effect of CD, two-implant-supported OD, and AO4 prostheses on bite force and masseter muscle activity during mastication of soft, medium, and hard food	Higher bilateral masseter muscle activity was observed for AO4 prostheses compared to two-implant-supported OD and CD. Additionally, the same outcome was found during the assessment of bite force.

Key Findings 

All three studies demonstrated higher masseter muscle EMG activity in AO4 prostheses than in other comparator CD or OD. In mono-arch AO4 studies, EMG amplitude ranked as milled-bar OD > fixed PFM > CD. Meanwhile, a bi-arch study showed a bilateral EMG increase and a 1.7-2.3-fold bite force increase over OD and CD across soft, medium, and hard food tests. In mono-arch AO4, provisional acrylic resin increased masseter EMG activity but produced greater occlusal contact imbalance than definitive PFM. Overall, bi-arch AO4 resulted in the greatest neuromuscular performance, followed by mono-arch AO4, OD, and CD. 

Discussion 

In this systematic review, we aimed to answer the PICO-formulated question: “How is the activity of the masseter muscle affected in fully edentulous patients rehabilitated with mono- and bi-arch AO4 prostheses?”. By synthesizing the three eligible clinical trials (n=37; 2020-2022), we found that both mono- and bi-arch AO4 prostheses significantly increase masseter EMG activity and bite force, with bi-arch designs demonstrating the greatest gains. 

Previous and contemporary research has shown that few clinical trials have focused on assessing the effects of mono- and bi-arch AO4 rehabilitation on the masseter muscle or the masticatory system in general, which may explain the absence of systematic reviews in the literature on this topic. However, across the findings of researchers who have contributed to this topic, similar observations and conclusions can be noted. These findings align with the widely accepted view that speech and chewing efficiency are solely dependent on masticatory muscle activity, which fundamentally relies on an individual’s dentition. Therefore, rehabilitating fully edentulous patients with mono- or bi-arch AO4 prostheses is expected to improve masticatory function progressively over time. Such notable changes are often reflected in the masseter muscle, due to its strategic anatomical position, and its ability to generate the highest bite force and chewing capacity compared to other masticatory muscles. However, the analysis of masticatory muscle activity was not limited to the masseter muscle alone, as some studies also included the temporalis and anterior digastric muscles in their assessments [26,28,29].

Based on this foundation, the first study was published by Dellavia et al. in 2012, in which bilateral surface EMG analysis of the masseter and temporalis muscles was conducted in patients with mono- and bi-arch AO4 prostheses, using dentate subjects as a control [28]. The results of static maximum clenching analysis revealed a balanced, symmetric muscle activity across all groups. Meanwhile, dynamic evaluations demonstrated the highest muscular activity and chewing coordination variability for mono-arch AO4 patients, followed by bi-arch AO4 subjects, with dentate individuals showing the least. Moreover, 80% of mono-arch and 50% of bi-arch AO4 patients presented with dominance of balancing-side muscle group activity and significantly less symmetrical chewing patterns [28].

In contrast, two years later in 2014, De Rossi et al. published the second study comparing bilaterally masseter and temporalis muscle activity in bi-arch AO4, full CD, and dentate groups [29]. The study revealed similar EMG muscle activity in both bi-arch AO4 and dentate subjects, with higher masseter activity than temporalis during clenching and non-habitual chewing, while CD users exhibited the opposite pattern. Meanwhile, habitual chewing demonstrated similar muscle activity across all three groups, with masseter activity exceeding that of the temporalis. At rest, the bi-arch AO4 and dentate groups maintained similar muscle activity levels, with dominant masseter activity, whereas CD patients exhibited significantly higher temporalis activity. Finally, muscular activity was reported to be symmetric across all assessments [29].

Complementing these results is the previously mentioned study by Soni et al., in which a bilateral analysis of masseter, temporalis, and anterior digastric muscles was conducted across three prosthetic types: CD, mandibular two-implant-supported OD, and bi-arch AO4 [26]. Their results consistently displayed the highest muscle activity in bi-arch AO4, followed by OD, and CD as the lowest. As expected, among the three muscle groups, the masseter muscle was the most active during mastication of all food consistencies, followed by the temporalis, and then the digastric. Nevertheless, no significant difference was observed between the right and left sides of the muscles [26]. 

Summarizing the findings of all the available studies, a consistent pattern of increased masseter, temporalis, and anterior digastric activity can be observed in patients rehabilitated with AO4, compared to those with two-implant-supported OD and CD, regardless of whether a mono- or bi-arch configuration was applied [26,28,29]. However, according to Dellavia et al., mono-arch AO4 demonstrated higher masseter and temporalis activity than both bi-arch AO4 and dentate patients. This was detected despite static assessments showing symmetrical muscle activity across all groups, while dynamic assessments revealed asymmetry [28]. 

In contrast, based on the results of De Rossi et al., masseter and temporalis activity in bi-arch AO4 patients was similar to that of dentate subjects, with symmetrical muscular coordination during both static and dynamic assessments [29]. Nevertheless, a possible factor contributing to asymmetrical neuromuscular coordination in mono- and bi-arch AO4 rehabilitated patients is the lateral mandibular displacement force that was mentioned by Dellavia et al., as both groups demonstrated values higher than dentate patients [28]. Interestingly, an equivalent phenomenon was observed in the study conducted by Aboelnagga et al., in which T-scan analyses of both acrylic resin provisional and PFM definitive mono-arch AO4 prostheses revealed imbalanced force distribution between the right and left sides, despite the insignificant correlation with muscle EMG activity [24]. 

Overall, the combined findings of the studies indicate greater muscle fatigue and restlessness in mono-arch AO4 patients compared to those with bi-arch AO4, possibly illustrating less competent neuromuscular adaptation and increased workload during mastication. A plausible explanation for the inconsistency of mono-arch AO4 prostheses compared to bi-arch AO4 and dentate patients may lie in the uniform rehabilitation plan used across all studies, in which a CD was placed in the maxilla and an AO4 prosthesis in the mandible. Therefore, as several studies [25,26,29] have illustrated the inferior masticatory performance of full CDs compared to other prosthetic options, a reduction of the overall efficiency in mono-arch AO4 rehabilitation would be expected.

In addition to specific muscle EMG analyses, several studies have evaluated the overall masticatory efficiency of fully edentulous patients rehabilitated with AO4 prostheses [30-32]. Elsyad et al. investigated masticatory efficiency and maximum bite force in patients using CD, FPD, and MBO prostheses for mono-arch AO4 rehabilitation [30]. The study concluded that both FPD and MBO prostheses used in AO4 significantly improved masticatory efficiency and maximum bite force, compared to CD [30]. Similarly, Denewar et al. compared the masticatory efficiency between two-implant-supported MBO and mono-arch AO4 prostheses, analyzing results based on gender and duration of edentulism [31]. Their study revealed better prognosis in the AO4 group, with notably greater efficiency in male patients and those with a shorter period of edentulism prior to prosthesis insertion [31]. In contrast, Jasser et al. performed a comparative assessment of masticatory efficiency in patients with either mono- or bi-arch AO4 and CDs using a gum color-mixing analysis test [32]. As expected, AO4 prostheses demonstrated significantly superior masticatory efficiency compared to CDs [32]. 

Consistently, the findings of these studies align with those of Soni et al., showing the significant advantage of AO4 prostheses in mastication when compared to CDs. Interestingly, Elsyad et al.’s bite force assessment of mono-arch AO4 FPD revealed values similar to those of the bi-arch AO4 denture in Soni et al.’s study [26,30]. Notably, Elsyad et al.’s mono-arch AO4 MBO showed the greatest bite force value [30]. These comparisons could suggest that the type of fixation used in AO4 prosthetic rehabilitation may play a crucial role in neuromuscular performance and masticatory strength.

Additionally, it is important to mention that both Elsyad et al. and Soni et al. used similar methodology for EMG amplitude analysis of the masseter muscle during chewing [25,26]. When comparing Elsyad et al.’s mono-arch and Soni et al.’s bi-arch AO4 findings, the bi-arch AO4 showed superior values compared to the mono-arch AO4 FPD. Nevertheless, mono-arch AO4 MBO demonstrated EMG values nearly equivalent to the bi-arch AO4, although the latter remained slightly superior. According to Elsyad et al., this difference can be attributed to the type of fixation used for the AO4 prosthesis [25,26]. However, another possible factor may be the material composition of the AO4 prostheses, as Aboelnagga et al. demonstrated a significant difference in masseter muscle activity between acrylic resin provisional and PFM definitive AO4 FPD [24].

With respect to the outcomes, several limitations exist within the available research, including small sample sizes; heterogeneous EMG procedures precluding pooled analysis; variable test foods and outcome measures; follow-up periods of less than 12 months; absence of dentate control groups in two studies; selective reporting concerns and potential publication bias; and an age range of 50-65 years, limiting generalizability. These methodological inconsistencies restrict direct comparisons and cross-study analysis. Therefore, future multi-center randomized clinical trials with standardized EMG protocols and longer follow-up periods are recommended.

To acknowledge these gaps, future studies should include larger sample sizes and a standardized EMG protocol. Moreover, evaluating long-term effects of mono- and bi-arch AO4 prostheses on the masseter muscle would provide valuable insights. Additionally, expanding the scope beyond the masseter to include muscles such as the pterygoideus and digastric would enhance our understanding of the neuromuscular impact of mono- and bi-arch AO4 prosthetic rehabilitation in fully edentulous patients.

## Conclusions

As the first systematic review on this topic, and despite its limitations, existing clinical trials have reported a higher masseter muscle activity in fully edentulous patients rehabilitated with either mono- or bi-arch AO4 prostheses compared to two-implant-supported ODs and CDs. However, bi-arch AO4 rehabilitation demonstrated superior neuromuscular performance of the masseter muscle compared to mono-arch AO4. Furthermore, the studies have illustrated that, regardless of whether a mono- or bi-arch configuration is used, several factors may influence the masseter muscle activity, such as the type of implant-prosthesis fixation and prosthesis material composition. Nevertheless, further high-quality research with bigger samples and longer follow-ups is required to draw more comprehensive conclusions regarding masseter muscle activity following mono- and bi-arch AO4 prostheses rehabilitation in fully edentulous patients.
